# The Oxidized Lipoproteins In Vivo: Its Diversity and Behavior in the Human Circulation

**DOI:** 10.3390/ijms24065747

**Published:** 2023-03-17

**Authors:** Hiroyuki Itabe, Takashi Obama

**Affiliations:** Division of Biological Chemistry, Department of Pharmaceutical Sciences, Showa University School of Pharmacy, 1-5-8 Hatanodai, Shinagawa-ku, Tokyo 142-8555, Japan

**Keywords:** oxidized LDL, oxidized HDL, Lp(a), electronegative LDL, sdLDL, oxidized phosphatidylcholine, cardiovascular diseases

## Abstract

A high concentration of low-density lipoproteins (LDLs) in circulation has been well-known as a major risk factor for cardiovascular diseases. The presence of oxidized LDLs (oxLDLs) in atherosclerotic lesions and circulation was demonstrated using anti-oxLDL monoclonal antibodies. The so-called “oxLDL hypothesis”, as a mechanism for atherosclerosis development, has been attracting attention for decades. However, the oxLDL has been considered a hypothetical particle since the oxLDL present in vivo has not been fully characterized. Several chemically modified LDLs have been proposed to mimic oxLDLs. Some of the subfractions of LDL, especially Lp(a) and electronegative LDL, have been characterized as oxLDL candidates as oxidized phospholipids that stimulate vascular cells. Oxidized high-density lipoprotein (oxHDL) and oxLDL were discovered immunologically in vivo. Recently, an oxLDL-oxHDL complex was found in human plasma, suggesting the involvement of HDLs in the oxidative modification of lipoproteins in vivo. In this review, we summarize our understanding of oxidized lipoproteins and propose a novel standpoint to understand the oxidized lipoproteins present in vivo.

## 1. Introduction

Atherosclerosis is a major pathological condition that can lead to cardiovascular diseases. Although multiple factors are involved in the onset and development of atherosclerosis, oxidative damage to lipoproteins has been thought to be implicated in the generation and progression of atherosclerosis and resulting cardiovascular diseases (CVD) [1,2].

As the major neutral lipids, triacylglycerol (TG) and cholesterol ester, are highly hydrophobic, they pack together with phospholipids and proteins to form complex particles dispersed in plasma. Lipoproteins are constituted by a core of TG and cholesterol ester surrounded by a phospholipid monolayer coupled with specific apolipoproteins, such as apolipoprotein B (apoB) or apolipoproteinA1 (apoA1). The lipoproteins act as carriers of cholesterol and TG in the circulatory system. Very low-density lipoproteins (VLDLs) deliver fatty acids to various peripheral tissues. Subsequently, the resulting low-density lipoproteins (LDLs) transfer cholesterol to peripheral tissues. Meanwhile, high-density lipoproteins (HDLs) remove cholesterol from the peripheral tissues and bring it back to the liver. HDL is well-recognized as an anti-atherogenic lipoprotein, as HDLs possess several beneficial functions such as endothelial cell protection and anti-oxidative and anti-inflammatory effects as well as reverse cholesterol transport [3,4]. In addition, epidemiologic data have shown that HDL-cholesterol levels correlate inversely with the occurrence of CVD [5]. 

The accumulation of a massive amount of cholesterol in vessel wall tissues is a typical feature of atherosclerosis. Macrophages that accumulate TG or cholesterol ester in atherosclerotic lesions are called “foam cells” because the number of large intracellular lipid droplets look like spongy foam. Foam cells are mostly derived from macrophages, however, other types of cells, such as endothelial cells and smooth muscle cells, also accumulate lipids to some extent. The LDL is the major cholesterol carrier in circulation, and the LDL concentration is mainly controlled by VLDL secretion from the liver and LDL receptor-mediated clearance from the circulation system. When the LDL is oxidatively modified, the resulting oxidized LDL (oxLDL) is no longer the ligand of the LDL receptor but binds to scavenger receptors. The expression of LDL receptors is regulated by the cholesterol content in cells, which protects cells from accumulating too much cholesterol. Macrophages and other cells take up oxLDL through scavenger receptors that are not down-regulated even when cells accumulate cholesterol, leading to foam cell formation. In addition, oxLDLs can stimulate vascular cells to induce inflammatory responses. Several clinical studies have shown that plasma oxLDL levels in patients with CVD are higher than that in control individuals [6]. 

**Figure 1:**
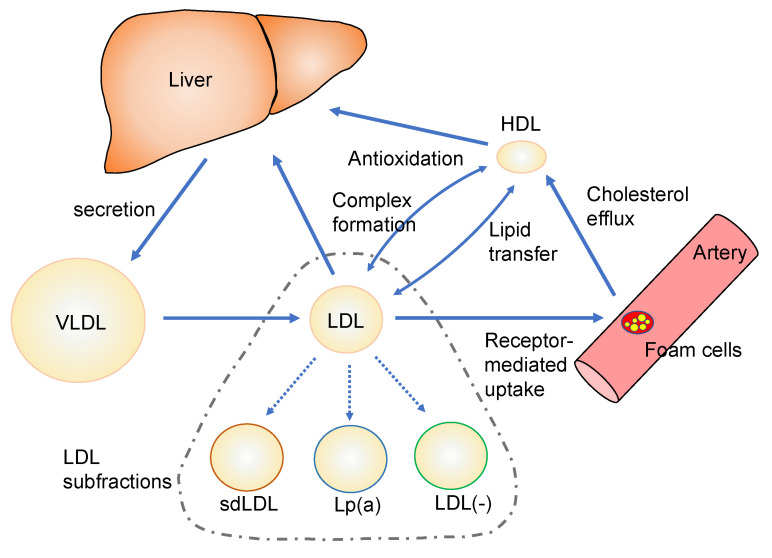
An illustrated diagram of the inter-relationship between lipoproteins and LDL subfractions. The dashed line includes LDL particles present in circulation. The three types of LDL subfractions and the function of HDL are described in Section 4. sdLDL: small dense LDL, Lp(a): lipoprotein small a, and LDL(-): electronegative LDL.

Enzyme-linked immunosorbent assay (ELISA) procedures to measure oxLDL levels became available in the 1990s which facilitated a wide range of clinical research on this issue [7]. The so-called “oxidized LDL hypothesis” has been strengthened by a number of studies, including the basic chemical analysis of the oxidation products generated from LDLs, the biological properties of oxLDLs, the metabolic behavior of oxLDLs in animal bodies, and clinical research on the relationship between oxLDL levels and diseased conditions. However, the actual figures of oxLDLs in vivo have yet to be fully elucidated. OxLDLs prepared in vitro mimic some properties of oxLDLs in vivo, however, copper-induced oxLDLs and well-studied oxLDLs in vitro, or their equivalent, are not present in circulation. Different ways to prepare oxLDL in vitro have been examined to better mimic oxLDLs in vivo, and LDL subfractions containing unique properties have been studied. In this review, we briefly summarize the characteristics of in vitro modified lipoproteins and LDL subfractions to propose updated features of in vivo oxLDLs (Figure 1).

## 2. OxLDL Prepared In Vitro

Copper-induced oxLDLs have been widely used as standard models of oxLDL since the 1990s and are currently being used because they are reproducible and easy to prepare. LDLs are heavily oxidized when an aliquot of LDLs (EDTA-free) is incubated with a low concentration of CuSO_4_ for several hours. Copper ions encourage effective LDL oxidation partly because copper ions bind to apoB. Lipids containing polyunsaturated fatty acyl chains and the side chains of the apoB protein are attacked by free radicals, and subsequently, they propagate radical chain reactions. The apoB polypeptide is cleaved by the radical chain reaction and the cross-linking of apoB fragments proceeds. Furthermore, lipid oxidation products, such as acrolein, malondialdehyde (MDA), 4-hydroxynonenal, and oxidized phosphatidylcholine (oxPC), covalently bind to the apoB protein to form various adducts.

However, copper-induced oxLDL is unlikely to occur in in vivo conditions based on two reasons. First, the metal ion-induced oxidation of LDL may be protected in plasma by high concentrations of plasma proteins and antioxidants [8]. Alternatively, the oxidation of LDLs by enzyme-mediated reactions has been investigated and may potentially proceed in the presence of plasma. Second, oxLDL particles enriched with oxPCs and heavily modified on their apoB protein moiety are readily cleared from the circulation [9]. Thus, oxLDL particles that are moderately modified would stay longer in vivo and would be detectable. 

In the earlier studies, different conditions of LDL oxidation were examined since the oxLDLs prepared by copper sulfate were thought to be severely modified. An alternative type of oxLDL called a minimally modified LDL (MM-LDL) was prepared with mild oxidation conditions and the process of dialysis [10]. MM-LDLs showed stimulatory responses on endothelial cells [11], although MM-LDLs showed only a slight increase in TBARS [12]. Dialysis during oxidative modification would remove the soluble products such as MDA and acrolein that are highly reactive to protein side chains, while reactions between hydrophobic molecules are able to proceed in the particles. Therefore, the protein modification by these small soluble molecules decreased in MM-LDLs compared with oxLDLs, and the surface charges on MM-LDLs became minimal. In contrast, oxPC molecules stayed in the modified LDL particles since they are not dialyzed easily. MM-LDL is thought to be a modified LDL enriched with oxPC [12]. When LDLs are oxidatively modified in vivo, LDL particles continuously interact with the surrounding water. Considering the dialyzing effect of small soluble products under physiological conditions, MM-LDL is likely to mimic some characteristics of in vivo oxLDL.

## 3. Detection of oxLDL In Vivo Using Immunological Methods

Although the expression of the LDL receptor is tightly regulated by the cholesterol content in the cells, the discovery of scavenger receptors explains how macrophages and other cells accumulate large amounts of lipids. In addition, oxLDL-induced foam cell formation could explain the mechanisms leading to the building up of lipid-rich, fragile atherosclerotic lesions that are indirectly related to CVD risks. Several scavenger receptors bind to oxLDL, including SR-A, CD36, and LOX-1 [13]. While these receptors are diverse in their protein structures, they recognize polyanionic particles, which are now considered receptors for pathogen-associated modified patterns (PAMPs) [14,15]. 

Then, an issue to be answered is what are the natural ligands of scavenger receptors responsible for foam cell formation? However, the presence of oxLDL in vivo was not proven at the time of the discovery of scavenger receptors. To detect oxLDL in pathological tissues, researchers have developed monoclonal antibodies (mAbs) bound to oxLDL but not to unoxidized LDL and detected oxLDL-related deposits in the atherosclerotic lesions of the human coronary artery and carotid artery and those in model animals [16,17,18,19,20]. The oxidation-specific epitopes of these mAbs are not fully elucidated, they bind to either oxidized phospholipids, adducts formed on amino acid side chains, or peptides in apoB that appear on the surface after oxidative modification. OxLDL was detected in the necrotic core enriched with macrophages in human atherosclerotic lesions, and in many cases, oxLDL antigens co-localized with foam cells derived from macrophages. The accumulation of oxLDLs in foam cells was supported by the observations that oxLDL was resistant to hydrolysis in lysosomes and that fragments of oxidized apoB accumulated in macrophage-derived foam cells in vitro [21]. 

These anti-oxLDL mAbs can be utilized to measure oxLDL in circulation by sandwich ELISA methods using two different antibodies; one antibody recognizes an oxidation-specific epitope and the other antibody recognizes apoB protein [7]. An interesting variation of oxLDL detection is a sandwich method using a recombinant protein of scavenger receptor LOX-1 and an anti-apoB mAb [22]. This strategy aims to detect any modified LDL particles that act as ligands of the scavenger receptor and is not limited to a narrow sense of oxLDL. Although the values for oxLDL levels in plasma calculated by the different methods cannot be compared directly, the increase in oxLDL levels in patients with CVD compared with control individuals was repeatedly observed [23]. A meta-analysis covering 12 studies using different procedures to measure oxLDL demonstrated the implication of circulating oxLDL in CVD [6]. The association of oxLDL with CVD was reported regardless of study designs, such as nested case-control or community-based cohort studies.

## 4. Characterization of Modified Lipoproteins and LDL Subfractions

Since the presence of oxLDLs in circulation was demonstrated, many studies have tried to characterize oxLDL, however, the actual features of oxLDLs present in vivo have not been fully elucidated. An LDL is a large particle consisting of one huge apoB protein (molecular mass over 500 kDa) and hundreds of lipid molecules. Furthermore, oxidative modification generates a wide variety of modified products and conjugates. Copper-induced oxLDL has been widely used as a standard and thought to mimic some characteristics of the in vivo oxLDL, however, such a heavily modified oxLDL was unlikely to be present in in vivo conditions, as described in Section 2. The complexity and metabolic behavior of in vivo oxLDL make it difficult to isolate it from the plasma and analyze its molecular structures. Although the oxLDL levels in plasma have been measured immunologically, it is not easy to isolate oxLDL from human plasma. Therefore, in vivo oxLDL has been a hypothetical material.

In the following subsections, three types of LDL subfractions and oxidized forms of other lipoproteins are discussed. Such information would be useful to elucidate the actual feature of oxLDL in vivo.

### 4.1. Small Dense LDL

Another approach to finding atherogenic lipoproteins is the fractionation of LDL. LDLs are defined as lipoprotein particles with a density between 1.019 and 1.063. However, they are not uniform in particle size, density, and composition but a mixture of heterogeneous particles. Austin et al. reported that LDLs could be separated into more than two subfractions with different diameters by non-denaturing gradient gel electrophoresis [24]. From the profile of LDL subfractions, two phenotypes with different LDL sizes were described; phenotype A was enriched with large, buoyant LDL particles, and phenotype B had a major peak of small, dense LDL particles. Small dense LDL (sdLDL) is a subfraction of LDLs with a diameter of less than 25.5 nm and a density between 1.044 and 1.063. Hirano et al. reported a conventional method to evaluate sdLDL levels in plasma using a heparin-magnesium precipitation procedure [25]. However, a commercially available, less labor-intensive application, the Lipoprint^®^ LDL system, is often used in clinical settings [26]. Increased sdLDL levels have been observed in patients with diabetes mellitus and coronary heart disease (CHD), and it is potentially a clinical marker for these diseased conditions [27,28]. 

It has been suggested that sdLDL is oxidatively modified. Several reports have shown that sdLDL is more susceptible to oxidative stress than large buoyant LDL [29,30]. The MDA-LDL level measured by a sandwich ELISA method was higher in the subjects with smaller LDL diameters [31]. When obese individuals were fed a moderate-fat diet supplemented with an avocado per day, or oleic acid oils to substitute the avocado, for 5 weeks, the change in the sdLDL number correlated with a change in the oxLDL level [32]. These observations suggest that sdLDL is related to oxLDL. However, there is little direct evidence to show oxidative modifications in isolated sdLDL particles.

### 4.2. Lipoprotein(a) 

**Figure 2:**
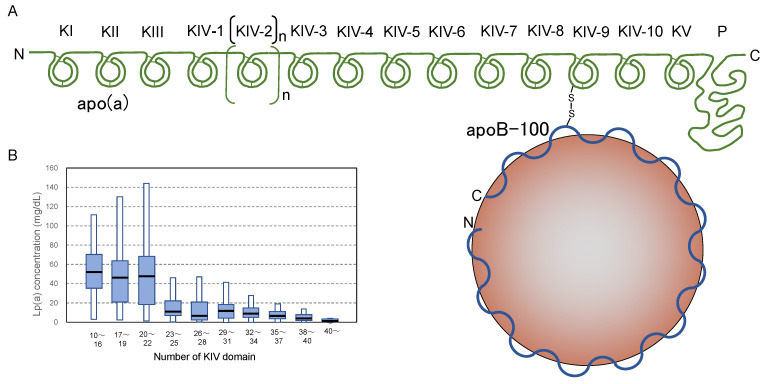
Structure and plasma concentrations of Lp(a). (**A**) An illustration of the protein structure of Lp(a). Lp(a) is constructed with an LDL and apolipoprotein(a), abbreviated as apo(a), bound by a disulfide bond. Apo(a) is a large protein consisting of several kringle domains and a protease-like domain (P) at the C-terminus. Kringle domains are numbered KI, KII, KIII, KIV-1 to KIV-10, and KV, where the KIV-2 domain repeats many times continuously. (**B**) The number of KIV repeats is defined genetically, and the plasma Lp(a) concentration in individuals is correlated with the number of KIV domains in the apo(a) protein. The box plot indicates the mean value (black horizontal line), 25–75% range (wide blue box), and estimated distribution range (white bar). The graph was modified from Figure 2C in the review article by Coassin and Kronenberg [36].

Lipoprotein(a) (Lp(a)) is a unique subfraction of LDLs which consists of an LDL particle and a large protein called apolipoprotein(a) (apo(a)). ApoB in the LDL particle and the apo(a) protein bind covalently through a disulfide bridge between the cysteine residues at the specific sites of both proteins (Figure 2A). Apo(a) is a soluble serum protein containing a protease-like domain at the C-terminus and multiple repeats of the kringle domains that are found in plasminogen. The kringle domains are classified as kringle I (KI) to kringle V (KV), and kringle IV contains 10 subtypes, kringle IV type 1 (KIV-1) to kringle IV type 10 (KIV-10). Since the KIV-2 domain repeats multiple times (from 1 to over 40 times), the number of kringle varies from 14 to more than 50. Thus, the molecular weight of apo(a) ranges from approximately 300 to 800 kDa [33]. The number of kringle repeats is defined individually by genetic variations, and the copy numbers of the KIV-2 sequence in the *LPA* gene vary among ethnic groups [33,34,35]. There is a tendency for the Lp(a) concentration to decrease as the number of kringle repeats increases. When the copy number of KIV-2 is less than 22, the average Lp(a) concentration is higher than 40 mg/dL and the variation is substantial (Figure 2B). Many SNPs on the *LPA* gene affect the Lp(a) concentration [36]. 

Tsimikas and colleagues found a close association between the oxPC-apoB adducts in LDL and Lp(a) fraction, and they demonstrated oxPC-bearing apoB particles as well as Lp(a) associated with CVD [37]. Interestingly, Lp(a) binds oxPC at the Asp57 residue in the kringle IV-10 domain in apo(a) covalently [38] so that Lp(a) can be detected by their sandwich ELISA system using an anti-oxPC mAb and an anti-apoB mAb. 

The possibility of the plasma Lp(a) concentration as a clinical marker has been extensively studied. A large-scale collaboration study of meta-analyses to assess Lp(a) as a risk factor for coronary heart disease (CHD) and based on 36 long-term prospective studies, recorded the Lp(a) concentrations of over 120,000 participants [39]. The study revealed that the risk of nonfatal myocardial infarction, coronary death, and ischemic stroke increased when the Lp(a) concentration increased to more than 20 mg/dL. The Lp(a) concentration did not change the risk of nonvascular death. Several other meta-analyses and systemic reviews concluded that the Lp(a) concentration had modest associations with the risk of CHD and related vascular diseases such as stroke and atrial fibrillation [40,41,42,43,44,45].

Genetic approaches to search SNPs related to vascular diseases have provided additional evidence to support the association between Lp(a) and vascular diseases. Clerke et al. reported a gene chip analysis to examine the association of Lp(a) genetic variants with the risk of CHD. DNA samples from more than 6000 participants were used to analyze 48,742 SNPs. Two variants at the *LPA* locus showed significant association with a reduced number of KIV-2 repeats, an increase in Lp(a) concentration, and an increased risk of CHD [46]. Klarin et al. conducted a genome-wide association study (GWAS) covering the datasets from more than 31,000 cases and 211,000 controls to search for genetic variants related to peripheral vascular diseases. Among the 19 loci related to peripheral artery disease (PAD), they discovered that an SNP of an intron variant of Lp(a) showed the lowest *p*-value and was associated with coronary diseases, carotid stenosis, and hypercholesterolemia [47]. A meta-analysis that covered 11 GWAS papers reported that two SNPs variants of Lp(a) were included in five loci correlated with peripheral vascular diseases [48]. Another GWAS using the eMERGE (Electronic Medical Records and Genomics) network revealed an association of a variant in *LPA* loci with the risk of coronary heart diseases and carotid artery atherosclerotic diseases [49]. The association between the *LPA* gene and the risk of vascular diseases has been further strengthened by Mendelian randomized studies [50,51,52,53]. 

It is noteworthy that, as described earlier, the distribution of Lp(a) concentrations differs by ethnic background [33,34,35], and that there are ethnic biases in Lp(a) implications in CVD risks. A 13-year follow-up study of a multi-ethnic cohort demonstrated that Lp(a) is related to the risk of heart failure only in white participants but was not significant in African, Hispanic, and Chinese participants [54]. Therefore, it has been questioned whether Lp(a) can be a universal marker. Many epidemiological studies, including the ones mentioned above, were performed on those with European ancestry. A GWAS study conducted on the Japanese population discovered three genetic variants associated with PAD. However, the *LPA* gene was not included [55]. 

The effect of lipid-lowering therapy on Lp(a) was evaluated by meta-analysis. Neither treatment with statin nor evolocumab, a PCSK9 inhibitor, affected Lp(a) concentration [56,57,58]. Although the catabolism of Lp(a) has not been fully elucidated, it has been considered that multiple receptors that recognize apoB, apo(a), or oxPC contribute to taking up Lp(a) into cells [59]. Both statins and PCSK9 inhibitors increase the LDL receptor-mediated catabolism of LDL. However, an increase in LDL receptors may not influence Lp(a) concentration, or only a little, if at all. Lp(a) concentration may depend on the apo(a) production rate which is genetically regulated.

### 4.3. Electronegative LDL

LDL particles are heterogeneous not only in their densities but in surface charges. The presence of an electronegative LDL subfraction in circulation has been known for several decades. Data have been accumulating on the unique characteristics of the electronegative LDL subfraction. 

**Figure 3:**
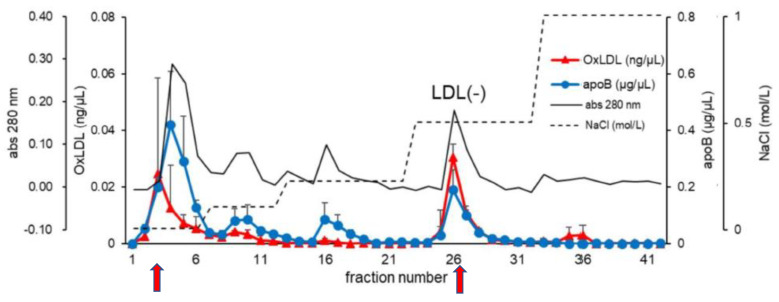
Chromatographic separation of LDL(−) and detection of in vivo oxLDL. LDLs recovered from patients with acute myocardial infarction were separated with anion-exchange column chromatography by step-wise elution with increasing NaCl concentration (dashed line). Elution profiles of apoB (blue circle) and oxLDL (red triangle) detected by sandwich ELISA systems were indicated. LDLs modified with oxPC were detected in two peaks (red arrows) at fractions 3–5 and 26–27. The last peak eluted with 0.5 M NaCl is the LDL(−) fraction. The graph is cited from Figure 1B in the review article by Sawada et al. [68] with slight modifications.

Cazzolato et al. reported a part of LDL absorbed on anion-exchange chromatography and separated from normal LDL; they called this electronegative LDL “LDL(−)” [60]. Later, Chen et al. fractionated LDL into five fractions on anion-exchange column chromatography and called the most electronegative fraction “L5” fraction [61]. Both LDL(−) and L5 describe the electronegative LDL fraction in the literature, however, they are comparative in their characteristics in general [62]. Therefore, the term LDL(−) is used in this review, henceforth (Figure 3).

The LDL(−) fraction accounts for approximately 4% (ranging from 0.5 to 9.8%) of all LDL, and it is characterized by the enrichment of TG, oxysterols and thiobarbituric acid-reactive substances, and lipid hydroperoxides but is poor in α-tocopherol [60,61,63,64,65]. LDL(−) showed stimulatory properties against endothelial cells to induce various cytokine expressions [66] and induce apoptosis of bovine endothelial cells in culture [60]. The LDL(−) may be mildly oxidized judging by the presence of lipid hydroperoxides and other products; however, Sánches-Quesada et al. reported no difference between LDL(−) and normal LDL concerning MDA, fatty acid hydroperoxides, and antioxidants [67]. Sawada et al. thoroughly characterized LDL(−) and found that LDL(−) was enriched with side chain oxidative modifications on apolipoproteins, but the concentration of oxPC was almost the same as normal LDL [68]. 

The LDL(−) fraction was found to be immunoreactive to an anti-oxPC mAb judging by a Western blot of LDL(-) suggesting that the apoB protein was covalently modified with oxPC [68]. However, the LDL(−) was not enriched with either lysoPC or free oxPCs that were extractable in the organic phase. In addition, the LDL(−) contained an almost equal amount of apoA1 and apoB (based on protein mass). Under electron microscopy, a close association of LDL and HDL particles was observed. Before the anion-exchange separation of LDL(−), LDL was first recovered in d = 1.019–1.63 fractions by sequential density ultracentrifugation, suggesting that the HDL in LDL(−) associated with LDL particles creates an apparent density less than 1.063. Through LC-MS/MS proteomic analysis, numerous oxidative modifications were observed on amino acid residues in apoA1 rather than apoB. Lp(a) was present in the LDL preparation before the anion-exchange chromatography, but it did not recover in the LDL(−) fraction. Most importantly, the amount of LDL(−) fraction recovered from patients with acute myocardial infarction was approximately three times higher than that from the control individuals [68]. The electronegative properties of LDL particles recovered from aortic tissues were described in an early report [69]. 

It is noted that an early study by Chappy et al. fractionated LDL into 10 fractions by HPLC, and the electronegative factions (L8–L10) did not show lipid peroxidation or the derivatization of amino acid residues of apoB [70]. This contradicts our observation because they used an LDL fraction pretreated with immunoaffinity chromatography to remove Lp(a) and apoA1-containing lipoproteins. 

When we separated an LDL(−) fraction with anion exchange column chromatography, an oxPC-positive LDL was also observed in an electroneutral LDL fraction that passed through the column, suggesting that in vivo oxLDL is not a single product, but that there are different types of oxLDL [67] (Figure 3). 

Furthermore, it is also suggested that HDL is profoundly involved in oxLDL generation in vivo conditions. Hazen and colleagues demonstrated the presence of oxHDL in circulation and atherosclerotic lesions [71,72]. As apoA1 is more susceptible to oxidative modification than apoB [73], it is possible that, during the oxidative modification of LDLs, neighboring active residues on the apoB protein can modify HDL. Consequently, apoB is protected from further modifications, but the oxLDL-oxHDL complex is potentially formed.

Data has been accumulating on the biological properties of LDL(−). LDL(−) was reported to induce cytokine release from THP-1 macrophages more than in vitro modified LDLs [74]. LDL(−) increased release of metalloproteinase-9 (MMP-9) and tissue inhibitors of metalloproteinase-1 (TIMP-1) from THP-1 macrophage through CD14 signaling [75]. LDL(−) was also reported to affect macrophage polarization to proinflammatory M1 phenotype [76]. LDL(−) was shown to induce foam cell formation of THP-1 cells to bind to various receptors, including LOX-1 and CD14, and LOX-1 is responsible for absorption into the macrophages [74,77].

Some studies tried elucidating the active molecules in LDL(−) fractions. As the accumulation of lipid hydroperoxides in LDL(−) was not prominent, other lipid metabolites were examined. Benitez, et al. reported that non-esterified fatty acids (NEFA) may be involved in endothelial stimulation because LDL(−) functions were reproduced either by the treatment of LDL with phospholipase A_2_ or by loading with NEFA [66]. By using inhibitors for sphingolipid metabolism, it was proposed that sphingosine in LDL(−) acted to induce inflammation, and ceramide and S-1-P were positive- and negative effectors of apoptosis [78]. Alternatively, to elucidate the roles of modified proteins in LDL(−), a mimetic peptide of LDL(−) was investigated as an inducer of inflammation reactions. Using 6- and 8-amino acid-long peptide libraries, a circular peptide of LDL(−) that binds to a monoclonal antibody to LDL(−) was discovered. This peptide, p2C7, activated NF-kB and inflammasome NLRP3 [79,80]. Interestingly, LDL(−) was found to form a complex with adiponectin in circulation, and adiponectin inhibited cellular responses by LDL(−), including NF-kB activation [81]. Recently, we found that the LDL(−) fraction was capable of enhancing the protrusion of neutrophil extracellular traps (NETs) from HL-60-derived neutrophils [82]. The efficacy of LDL(−) for enhancing NET formation was slightly weaker than that of copper-induced LDLs. However, this would be explained by LDL(−) containing a lower amount of free oxPC and lysoPC than copper-induced oxLDL.

Several clinical studies have elucidated the relationship between LDL(−) and CVD risks. Chu et al. reported that the concentration of LDL(−) and the percent LDL(−) in total LDL in patients with CHD were significantly higher than that in healthy adults [83]. The same group also retrospectively analyzed the hyperlipidemic patients who received statin therapy for three months. When the patients were classified into statin-benefit and non-statin-benefit groups, the statin-benefit group, who are expected to reduce their risks of CVD through statins, had a significantly higher concentration of LDL(−) and the percent of LDL(−) in total LDL compared to non-statin benefit group [84]. LDL(−) levels were also higher in hemodialysis patients with lower extremity PAD than in hemodialysis patients without PAD [85]. In addition, a number of studies showed increased LDL(−) values in patients with familial hypercholesterolemia and diabetes mellitus [86].

A new strategy to trap LDL(−) using a chitosan-based nano particle conjugated with a single-chain variable fragment of anti-LDL(−) mAb was developed. This nanoparticle was taken up by human and mouse macrophages via macro-pinocytosis, and when this nano-particle was injected intravenously into LDL receptor knockout mice the inflammatory responses in the mice and the aortic lesion size were significantly reduced [87]. Although this nano-particle has yet to be examined, this is an exciting approach to minimize the pathologic property of LDL(−).

### 4.4. Oxidatevely Modified HDL

HDLs are susceptible to oxidative modification as in the case of LDLs. HDLs are readily oxidized by incubation with copper ions, radical generators, hypochlorous acid, or myeloperoxidases in vitro [88,89,90,91]. Interestingly, a variety of oxidative modifications of HDLs were induced by incubation with oxLDL [89] or oxPC [73,92] which mimics physiological conditions, indicating that the modification of apoA1 occurs simply with preformed oxidized products. OxHDLs including oxPC-apoA1 adducts and chlorinated or carbamylated apoA1, were detected in human circulation and atherosclerotic lesions [72,93,94]. 

HDLs have been thought to be athero-protective through the reverse cholesterol transport function and anti-oxidant properties [95]. In addition, the interaction of HDLs with lipid oxidation products in oxLDLs would be an essential function of HDLs. The co-incubation of HDLs protected LDLs from further oxidation by reducing phospholipid hydroperoxides in LDLs [96]. The HDL receives oxPC molecules transferred from oxLDL particles, and the oxPC is subsequently metabolized to diacyl- non-oxidized PC [89,97]. The cholesterol efflux capacity of HDLs is severely affected by the oxidative modification of specific sites of apoA1 [91]. The interaction between lipoproteins should be inevitable in circulation and a complex of oxLDL and oxHDL can be formed under physiological conditions [98]. These observations suggest that HDLs prevent the further oxidation of LDLs through interaction with oxLDLs, while the HDL itself becomes modified and loses some of its athero-protective functions. 

To date, several procedures of sandwich ELISA for oxHDL determination in human circulation have been utilized to show the positive correlation between an increase in plasma oxHDL levels with CVD and metabolic mal-conditions [99,100,101]. In addition, HDLs recovered from patients with CHD induced the increased expression of proinflammatory cytokines and miRNA in endothelial cells [102,103]. The clinical implication of oxHDL is yet to be elucidated, and further study is needed to understand the relationship among the circulating levels of HDLs, LDLs, oxHDLs, oxLDLs, and other modified lipoproteins. As oxHDLs are likely to be generated by interaction with oxLDLs or any other oxidants, increased oxHDLs may be the result of the active antioxidant function of HDLs; alternatively, increased oxHDL levels could represent the overall stress in the body that HDLs need to interact [97]. 

## 5. Possible Perspective of oxLDL Generation and Metabolism

Recent progress regarding modified LDL and LDL subfractions would provide a better understanding of oxLDLs in vivo. We propose the following key points of consideration on in vivo LDLs:

1. In vivo oxLDLs can be heterogeneous and the extent of modifications can be gradual. In addition, the type of modifications can be multiple, and many different methods to generate in vivo oxLDLs may be possible.
2. LDLs are oxidatively modified in physiological circumstances, either in circulation or arterial tissues. Under the circumstances, other lipoproteins, including HDLs, are always present together with LDLs. Thus, oxLDLs could come into contact with other lipoproteins, and oxidized lipids could transfer from oxLDLs to other lipoproteins. In addition, the interaction of oxLDLs with various cells is also possible.
3. OxLDLs are cleared from circulation when they are heavily modified enough to bind to scavenger receptors. It is well known that heavily oxidized LDLs are rapidly cleared through liver Kupffer cells. Alternatively, macrophages in vascular tissues take up heavily modified LDLs and degrade them in the lysosomes. Thus, the concentration of heavily modified oxLDLs in circulation is substantially low. However, the oxLDL concentration could be variable depending on the types of modifications.
4. Oxidative modifications of LDLs and other lipoproteins occur constantly, and at the same time, the reduction of oxidized products and degradation of oxLDLs continues, and heavily modified LDLs are cleared. Thus, the oxLDL levels in circulation should be defined by the balance between the oxidative stress that produces oxLDLs and the protection, catabolism, and clearance of oxLDLs that decrease the oxLDL level; an increase in the plasma oxLDL could represent the imbalance between them.

**Figure 4:**
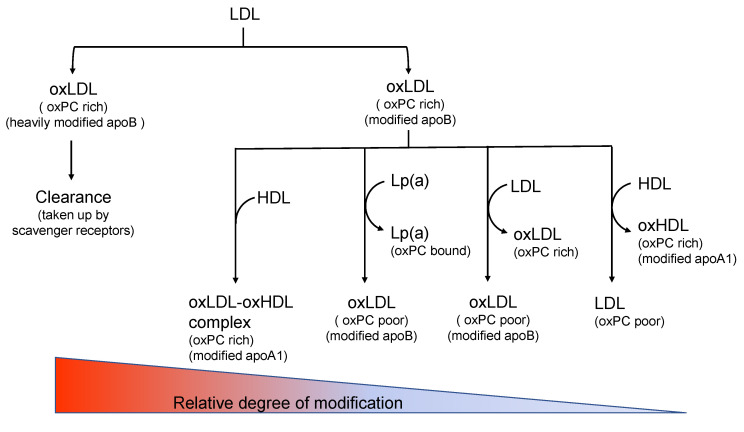
Possible interaction of lipoproteins during oxLDL generation. In in vivo circumstances, oxLDLs would interact with various lipoproteins such as LDL, HDL, and Lp(a). They would transfer some of the oxidized lipid products and change oxidized moieties on amino acid residues. They may also form an oxLDL-oxHDL complex. When an oxLDL is heavily modified, it will be cleared from circulation.

Considering the aforementioned points, we propose a possible explanation for the features of oxLDLs in vivo as follows (Figure 4):

During the oxidation of LDLs, lipids and the side chains of the apoB protein are oxidized by radical chain reactions. Lipid oxidation products containing reactive aldehydes bind covalently to apoB proteins to form adducts. Heavily modified oxLDL particles enriched with oxPCs and heavily modified apoB protein moiety are readily cleared from circulation. OxLDL particles that are moderately modified stay longer in circulation and interact with other lipoproteins, including HDLs, LDLs, or Lp(a). HDLs could receive oxPC molecules from oxLDLs and reduce oxidized amino acid residues in the apoB of oxLDLs. Subsequently, the oxLDL may minimize its oxidation properties. Alternatively, HDLs turn into oxHDLs and form complexes with oxLDLs. LDL particles could receive oxPC molecules from oxLDLs, and subsequently, LDLs would change to oxPC-rich oxLDLs and the oxLDLs would become oxPC-poor but rich in apoB modifications. Lp(a) could receive oxPC from an oxLDL to form oxPC-bound Lp(a), and the oxLDL would change to an oxPC-poor oxLDL. SdLDLs are formed as a consequence of hypertriglyceridemia. However, if sdLDLs stay in circulation longer, they can accumulate more oxPCs and apoB modifications than normal LDLs. It is likely that various types of oxidized lipoproteins, namely oxLDLs, oxHDLs, oxLDL-oxHDL complexes, oxPC-bound Lp(a), and possibly other forms, coexist under physiological conditions. 

**Figure 5:**
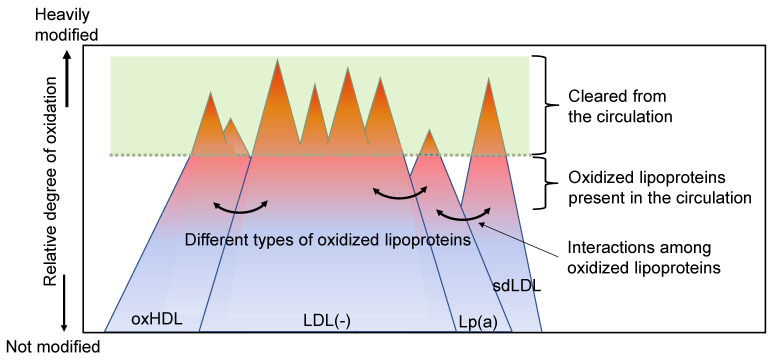
Proposed features of in vivo oxLDL. OxLDLs are a mixture of heterogenous types of modified lipoproteins. Lipoproteins interact under physiological circumstances and various lipoproteins are generated which are either not modified or moderately modified. When these lipoproteins are heavily modified, such particles are rejected from circulation. Thus, a part of modified lipoproteins that can stay longer in circulation can be detected.

Depending on the degree of protein modifications and the content of oxPC molecules in the lipoprotein particles, some of these modified lipoproteins behave as LDL(−). Similarly, depending on the composition of lipids and other factors, a part of the modified lipoproteins is classified into an sdLDL. Although the LDL(−) fraction would be enriched with modified lipoproteins, it is cautioned that this fraction could be heterogeneous and contain less modified LDL particles to some extent. Similarly, Lp(a) could carry oxPCs but a part of Lp(a) would be oxPC-free. In addition, different forms of modified lipoproteins coexist simultaneously in vivo, and they interact with each other (Figure 5). Oxidized lipoproteins are generated even under physiological conditions. Interactions between various lipoproteins could protect, metabolize, and reduce oxidative modifications. When they are heavily modified, such particles are cleared from circulation. Therefore, the oxLDL levels (or oxHDL levels) can reflect the total strength of oxidative stress, including the generation of oxidants responsible for oxidation reactions, protection of lipoprotein modification by other particles, and the clearance rate of modified lipoproteins.

## 6. Conclusions

While the oxLDL level has been well recognized as a risk factor for atherosclerosis and a potential biomarker for CVD, its actual figure has not been long clarified. The characterization of many types of modified lipoproteins and lipoprotein subfractions in plasma has been conducted for decades. Through these studies, a novel standpoint can be proposed for understanding the actual feature of oxidized lipoproteins in vivo. In physiological circumstances, the oxidative modification of LDLs and interaction between oxLDLs and other lipoproteins could occur constantly; LDL subfractions, Lp(a), LDL(−), and sdLD, can be oxidatively modified. Levels of oxLDL could represent the balance between the oxidative stress that generates oxLDL and the reduction, metabolism, and clearance that decrease oxLDL levels. Further studies on oxidized lipoproteins in vivo based on the molecular analysis of modified structures would elucidate their actual features.
